# Multi-Site Observational Study to Assess Biomarkers for Susceptibility or Resilience to Chronic Pain: The Acute to Chronic Pain Signatures (A2CPS) Study Protocol

**DOI:** 10.3389/fmed.2022.849214

**Published:** 2022-04-25

**Authors:** Giovanni Berardi, Laura Frey-Law, Kathleen A. Sluka, Emine O. Bayman, Christopher S. Coffey, Dixie Ecklund, Carol G. T. Vance, Dana L. Dailey, John Burns, Asokumar Buvanendran, Robert J. McCarthy, Joshua Jacobs, Xiaohong Joe Zhou, Richard Wixson, Tessa Balach, Chad M. Brummett, Daniel Clauw, Douglas Colquhoun, Steven E. Harte, Richard E. Harris, David A. Williams, Andrew C. Chang, Jennifer Waljee, Kathleen M. Fisch, Kristen Jepsen, Louise C. Laurent, Michael Olivier, Carl D. Langefeld, Timothy D. Howard, Oliver Fiehn, Jon M. Jacobs, Panshak Dakup, Wei-Jun Qian, Adam C. Swensen, Anna Lokshin, Martin Lindquist, Brian S. Caffo, Ciprian Crainiceanu, Scott Zeger, Ari Kahn, Tor Wager, Margaret Taub, James Ford, Stephani P. Sutherland, Laura D. Wandner

**Affiliations:** ^1^Department of Physical Therapy and Rehabilitation Science, Carver College of Medicine, University of Iowa, Iowa City, IA, United States; ^2^Department of Biostatistics, College of Public Health, University of Iowa, Iowa City, IA, United States; ^3^Department of Physical Therapy, St. Ambrose University, Davenport, IA, United States; ^4^Department of Psychiatry, Rush University Medical Center, Chicago, IL, United States; ^5^Department of Anesthesiology, Rush University Medical Center, Chicago, IL, United States; ^6^Department of Orthopaedic Surgery, Rush University Medical Center, Chicago, IL, United States; ^7^Departments of Radiology, Neurosurgery, and Bioengineering, University of Illinois College of Medicine at Chicago, Chicago, IL, United States; ^8^NorthShore Orthopaedic and Spine Institute, NorthShore University HealthSystem, Skokie, IL, United States; ^9^Department of Orthopaedic Surgery and Rehabilitation Medicine, The University of Chicago, Chicago, IL, United States; ^10^Department of Anesthesiology, University of Michigan, Ann Arbor, MI, United States; ^11^Department of Medicine (Rheumatology), University of Michigan, Ann Arbor, MI, United States; ^12^Department of Psychiatry, University of Michigan, Ann Arbor, MI, United States; ^13^Department of Psychology, University of Michigan, Ann Arbor, MI, United States; ^14^Section of Thoracic Surgery, University of Michigan, Ann Arbor, MI, United States; ^15^Section of Plastic and Reconstructive Surgery, University of Michigan, Ann Arbor, MI, United States; ^16^Department of Obstetrics, Gynecology and Reproductive Sciences, University of California, San Diego, La Jolla, CA, United States; ^17^Institute of Genomic Medicine Genomics Center, University of California, San Diego, La Jolla, CA, United States; ^18^Department of Internal Medicine, Wake Forest School of Medicine, Winston-Salem, NC, United States; ^19^Department of Biostatistics and Data Science, Wake Forest School of Medicine, Winston-Salem, NC, United States; ^20^Department of Biochemistry, Wake Forest School of Medicine, Winston-Salem, NC, United States; ^21^West Coast Metabolomics Center, University of California, Davis, Davis, CA, United States; ^22^Environmental and Molecular Sciences Laboratory, Pacific Northwest National Laboratory, Richland, WA, United States; ^23^Department of Pathology, University of Pittsburgh, Pittsburgh, PA, United States; ^24^Department of Biostatistics, Johns Hopkins Bloomberg School of Public Health, Baltimore, MD, United States; ^25^Texas Advanced Computing Center, The University of Texas at Austin, Austin, TX, United States; ^26^Presidential Cluster in Neuroscience, Department of Psychological and Brain Sciences, Dartmouth College, Hanover, NH, United States; ^27^Geisel School of Medicine at Dartmouth, Hanover, NH, United States; ^28^National Institute of Neurological Disorders and Stroke, The National Institutes of Health, Bethesda, MD, United States

**Keywords:** postsurgical pain, thoracic surgery, pain, biomarker, risk factors, protocol, knee arthroplasty

## Abstract

Chronic pain has become a global health problem contributing to years lived with disability and reduced quality of life. Advances in the clinical management of chronic pain have been limited due to incomplete understanding of the multiple risk factors and molecular mechanisms that contribute to the development of chronic pain. The Acute to Chronic Pain Signatures (A2CPS) Program aims to characterize the predictive nature of biomarkers (brain imaging, high-throughput molecular screening techniques, or “omics,” quantitative sensory testing, patient-reported outcome assessments and functional assessments) to identify individuals who will develop chronic pain following surgical intervention. The A2CPS is a multisite observational study investigating biomarkers and collective biosignatures (a combination of several individual biomarkers) that predict susceptibility or resilience to the development of chronic pain following knee arthroplasty and thoracic surgery. This manuscript provides an overview of data collection methods and procedures designed to standardize data collection across multiple clinical sites and institutions. Pain-related biomarkers are evaluated before surgery and up to 3 months after surgery for use as predictors of patient reported outcomes 6 months after surgery. The dataset from this prospective observational study will be available for researchers internal and external to the A2CPS Consortium to advance understanding of the transition from acute to chronic postsurgical pain.

## Introduction

Chronic pain is a significant health problem with 20% of Americans reporting moderate to severe pain and 25 million Americans reporting daily pain (1, 2). Further, chronic pain produces the largest non-fatal burden of disease as many individuals experience moderate-to-severe chronic pain that contributes to years lived with disability (3–5). While concerted efforts to better manage pain have occurred over the past two decades, the adverse sequelae of increased use and misuse of opioid treatment has brought this important issue to the forefront. Yet, our understanding of factors that contribute to the transition from an acute pain event to chronic pain are poorly understood, likely due to its complex nature and the inherent individual variability. Factors associated with the transition to chronic pain have been identified in animal and human studies, providing potential candidates for biomarkers, but few large-scale prospective studies have been completed to explore the predictive power of these biomarkers. Factors across biopsychosocial domains have been implicated including psychosocial factors such as anxiety, depression, resilience, social support, childhood and adult trauma (6–27), neuroimaging signals (28–32), quantitative sensory testing (33–39), molecular changes in genes, proteins, extracellular RNA, lipids, and metabolites (40–50), and multiple patient reported outcome measures such as pain intensity, sleep dysfunction, and disability (6, 13–15, 19, 20, 51–63). To that end, the Acute to Chronic Pain Signatures (A2CPS) Program was funded by the National Institutes of Health (NIH) Common Fund to identify biomarkers and their collective biosignatures (a combination of several individual biomarkers) that predict susceptibility or resilience to the development of chronic pain after an acute pain event. This goal will be accomplished by characterizing the predictive nature of multiple targeted primary, secondary, and exploratory biomarkers for identifying those who develop chronic pain 6 months after a surgical intervention with the following objectives.

Objective 1 will use a candidate approach to examine whether individual biomarkers with prior evidence predict susceptibility or resilience to the development of chronic pain.

Objective 2 will develop biosignature(s) using the candidate biomarkers to determine if combinations of biomarkers improve the prediction from acute to chronic pain.

Objective 3 is exploratory and will use a discovery-validation approach to define novel putative biomarkers without sufficient preliminary data and biosignatures with combinations of novel and candidate biomarkers that predict the susceptibility and resilience to development of chronic pain.

When selecting biomarkers, the Consortium considered several factors including scalability, clinical usefulness, and the current evidence. While the FDA-NIH Biomarker Working Group defines biomarkers as molecular, histologic, radiographic, or physiologic characteristics that provide an indication of biological or pathogenic processes (64), use of the term “biomarker” and “biosignature” in this manuscript incorporates markers across biological, psychosocial, and clinical domains for the indication of chronic post-surgical pain. Because pain is a multidimensional construct, A2CPS will incorporate a comprehensive list of biomarkers across the biopsychosocial spectrum to follow a biopsychosocial approach to pain (16, 65). Content experts within and outside the consortium reviewed candidate biopsychosocial constructs and selected markers with the strongest prior evidence for inclusion as primary and secondary biomarkers for A2CPS. The A2CPS program will capitalize on recent scientific advances and current knowledge in brain imaging, high-throughput screening techniques (omics), quantitative sensory testing (QST), patient-reported outcome (PRO) assessments, and functional assessments. Biomarkers and biosignatures, collected before the transition to chronic pain, have the potential to allow providers to identify high- vs. low-risk individuals, which could be a game changer in the prevention and treatment of chronic pain. The investigation of multiple candidate markers simultaneously in a relatively large cohort has the greatest potential to identify key predictors. The dataset will also serve as a resource for the research community to explore additional scientific inquiries.

Two study populations were selected for inclusion in A2CPS: individuals undergoing knee arthroplasty or thoracic surgery. These two clinical populations are exposed to an acute painful event (surgery) and will be followed to identify who transitions to chronic pain at 6 months following surgery. However, they differ in the type of and underlying causes requiring surgery as well as in baseline pain status: knee osteoarthritis typically presents with pre-surgical pain, whereas thoracic surgery typically does not. This design will allow for investigation of baseline biomarkers that predict susceptibility or resilience to chronic post-surgical pain in people already experiencing persistent pain (knee arthroplasty) and another without (thoracotomy). Chronic post-surgical pain (CPSP) is a major cause of suffering and disability, occurring at rates between 10 and 40% after common surgical procedures (66–70). More specifically, transition rates for CPSP following knee replacement have been reported to range from 10 to 30% (71–74), whereas roughly 30-47% of patients following thoracic surgery procedures develop new chronic pain after 6 months (75–80). Thus, these two populations provide the opportunity to identify unique and shared biomarkers and biomarker signatures that predict the development of or resilience to CPSP. This report provides an overview of the A2CPS prospective observational study protocol and summary of the general data collection procedures.

## Methods and Analysis

### Study Design

**Figure 1:**
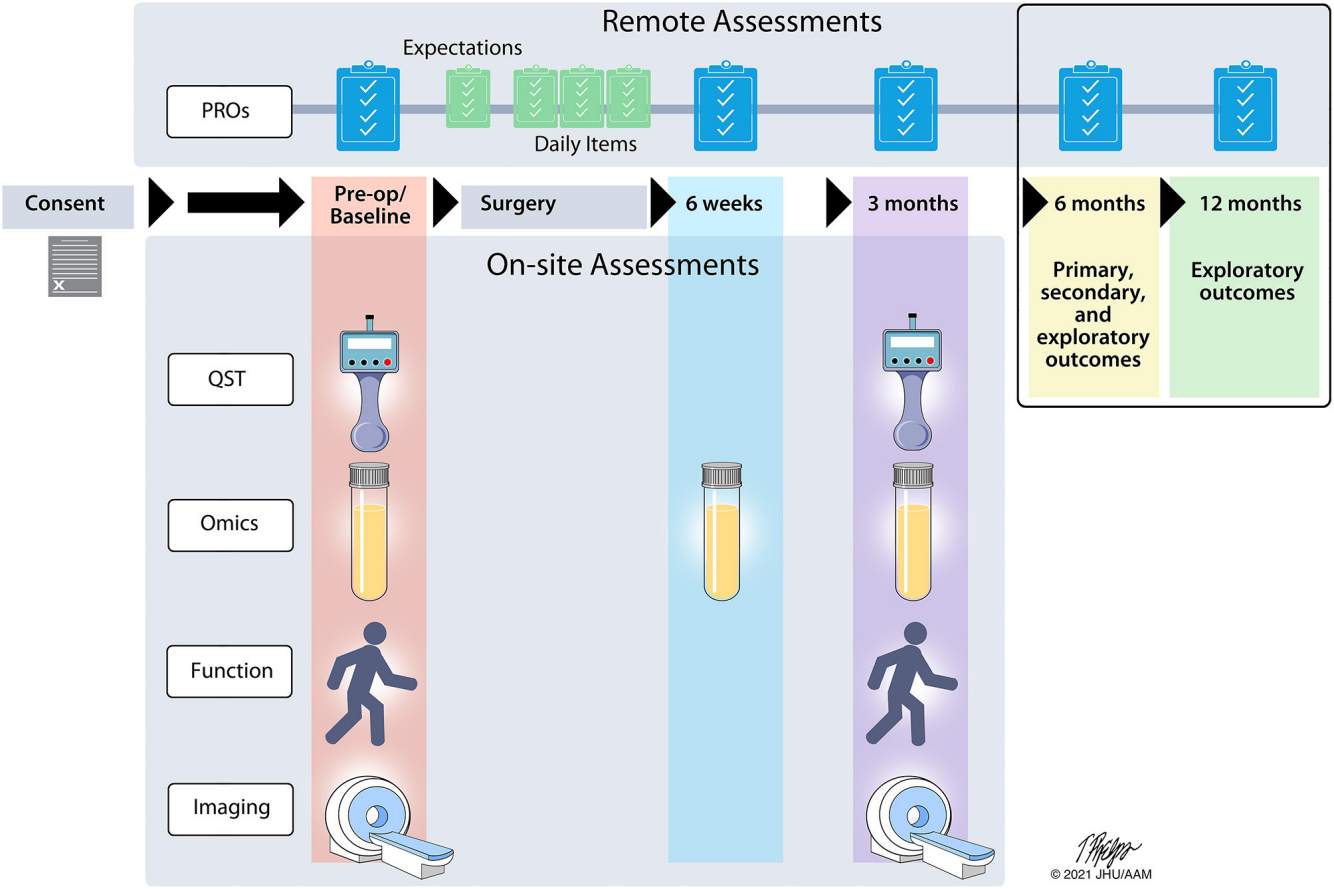
A2CPS study timeline. The flow of assessments is indicated across the 12-month study timeline. Remote assessments consist of patient reported outcomes (PRO) while onsite assessments involve quantitative sensory testing (QST), a blood draw for high-throughput screening techniques (omics), physical function, and brain imaging.

This is a longitudinal, multi-site, prospective observational study to identify candidate and novel biomarkers and biosignatures that predict development of or resilience to chronic pain 6 months after surgery. Multiple assessments of targeted PROs, QST, functional assessments, brain imaging, and biospecimen omics biomarkers will be assessed pre-surgery (baseline), across a 6-week post-operative period (psychosocial assessments and biospecimen only), and at 3 months (Figure 1) using a combination of on-site and remote assessments. All subjects will be asked to complete the baseline, acute post-operative, and 3-month assessments. The primary, secondary, and exploratory study endpoints will be assessed remotely at 6 months. In addition, subjects will be contacted at 12 months to collect exploratory follow-up endpoints. The selection and implementation of study assessments was completed with feedback from an advisory panel of content experts in each domain and two patients who previously underwent knee arthroplasty and thoracic surgery.

### Study Organization and Settings

**Figure 2:**
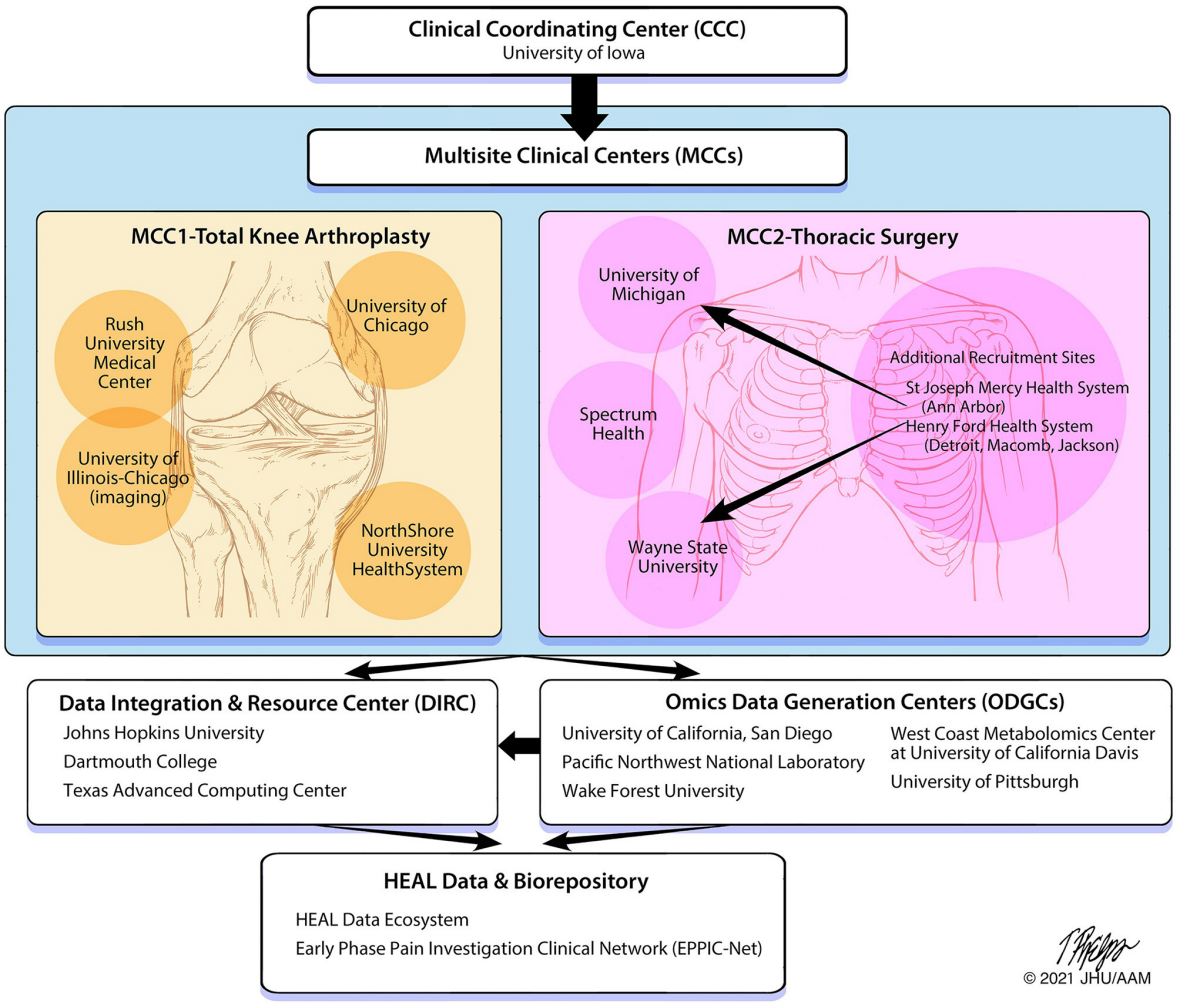
A2CPS structure and data flow. The organization of the A2CPS Consortium includes oversight from the Clinical Coordinating Center, data collection from the Multisite Clinical Centers, data generation from the Omics Data Generation Centers and Data Integration and Resource Center and storing of data and biospecimens within a repository.

The A2CPS Consortium consists of multiple sites/institutions with specified roles to fulfill study objectives (Figure 2), which includes two Multisite Clinical Centers (MCCs), a Clinical Coordinating Center (CCC), three Omics Data Generating Centers (ODGCs), and a Data Integration and Resource Center (DIRC). In addition to sites/institutions, four NIH-appointed external Program Consultants and a patient representative have been incorporated on study committees and provide feedback on study design and ongoing review of the Consortium. A Steering Committee is the main governing board for the Consortium and has leadership representation from each institution and the NIH. A2CPS is a longitudinal study with anticipated recruitment of 3600 individuals divided between two cohorts scheduled for knee arthroplasty or thoracic surgery. Each Multisite Clinical Center (MCC) is set up to successfully recruit the target sample size with enrolment of knee arthroplasty patients initiated at MCC1 and the thoracic surgery cohort at MCC2. Both MCCs have the capacity to enroll both patient cohorts if needed to achieve enrolment goals. Additionally, clinical sites which serve various socioeconomic, racial, and ethnic patient populations were selected for inclusion in both MCCs to increase the diversity of patient enrolment.

MCC1 is located in the Chicago metropolitan area with recruitment sites at Rush University, the University of Chicago and NorthShore University HealthSystem. These sites are all part of the Institute of Translational Medicine (81), funded by the University of Chicago/Rush University Clinical and Translational Science Award of the National Center for Advancing Translational Sciences (NCATS) which is one of the 27 institutes and centers of the National Institutes of Health. The University of Illinois at Chicago is also a partner in this Consortium bringing expertise in brain imaging research (82). MCC1 initiated recruitment of the knee arthroplasty population, where collectively, their sites perform more than 4,700 knee arthroplasty procedures annually. MCC1 also serves a diverse population of patients with more than 30% African American and 15% of Hispanic origin. Potential participants will be recruited from the practices of more than 15 adult reconstructive orthopedic surgeons, and the MCC1 site has the advantage of recruiting from multiple high-volume joint replacement centers.

MCC2 consists of recruitment sites from across the State of Michigan, performing all assessments at 3 testing sites: University of Michigan in Ann Arbor, Wayne State University in Detroit, and Spectrum Health in Grand Rapids. Additional health centers will fill in as recruitment pools with follow up testing at the 3 centers. MCC2 initiated recruitment of the thoracic surgery population. Potential participants will be recruited from 10 clinical sites who partner in the Michigan Society of Thoracic and Cardiovascular Surgeons (MSTCVS) Quality Collaborative (83).

The CCC is housed at the University of Iowa and includes a partnership between pain scientists from the Department of Physical Therapy and Rehabilitation Science in the Carver College of Medicine and clinical trialists from the Clinical Trials Statistical and Data Management Center (CTSDMC) in the College of Public Health. The CCC leads development and maintenance of the Master Protocol, the Manual of Procedures (MOP), and Standard Operating Procedures (SOPs); and is responsible for the central Institutional Review Board (cIRB) application and associated reliance agreements. To ensure consistent study design, progress and quality, the CCC works closely with the MCCs to provide training and monitoring of study procedures and assist with recruitment and retention procedures. A2CPS training is provided by the CCC to all sites to facilitate standardization of all study procedures and to improve data quality. Research assistants must complete a comprehensive training and certification process in administering and recording of data with inter-rater reliability checks prior to and every 6 months following site activation. Thus, the CCC ensures that processes are in place to allow the MCCs to collect high-quality data.

There are three ODGCs that each focus on different omics analyses. These ODGCs are responsible for preparing biospecimen-collection kits, analyzing samples collected by MCCs, and sending data to the DIRC for storage and analysis. The *ODGC—Genetic Variants and exRNA* is housed at the University of California, San Diego. *ODGC—Proteomics* is a collaboration between Pacific Northwest National Laboratories (PNNL) and the University of Pittsburgh and will analyze samples for proteins and cytokines. *ODGC—Lipidomics and Metabolomics* is a collaboration between the Center for Precision Medicine at the Wake Forest School of Medicine and the West Coast Metabolomics Center at the University of California, Davis.

The DIRC, primarily housed at Johns Hopkins University with collaborating sites at Dartmouth College and the Texas Advanced Computing Center (TACC), combines biostatisticians, informaticians, and database experts with pain scientists into a single integrated team. The DIRC integrates efforts of all funded components of the Consortium and serves as a community-wide nexus for protocol, assay and data standards. The DIRC set up data-processing pipelines for the imaging and omics data sets and a data-collection system (REDCap/My Data Helps) for data collected at the MCCs. The DIRC will perform statistical analysis of data to test the aims of the Consortium. The DIRC also leads an outreach component which includes a public website (www.a2cps.org), a portal for Consortium members, and will provide user-friendly, publicly accessible data to the scientific community for novel discovery approaches.

### Cohorts and Recruitment

Two different surgical cohorts are being recruited for A2CPS. The first cohort consists of individuals scheduled for unilateral knee replacement. The second cohort consists of individuals scheduled for thoracic surgery [thoracotomy, video-assisted thoracic surgery (VATS), and robotic thoracic surgery]. Patients are recruited by study research assistants at each clinical/recruitment site with assistance from clinic and study personnel. Collaborating surgeons and clinical staff provide information pertaining to study participation, but all screening and written informed consent is performed by dedicated A2CPS research assistants. The established cohorts have shared and cohort-specific eligibility criteria (Table 1). Patients are not excluded for prior chronic pain conditions, including fibromyalgia, neuropathic pain, peripheral neuropathy, low back pain, mental health conditions, or prior surgery > 3 months previously; or based on prior or baseline pain medication use, including opioids, to maximize generalizability. A comprehensive list of co-morbidities are documented in the preoperative (“baseline”) period. Study enrollment commenced with English-speaking patients, and Spanish-speaking patients are now eligible for participation. Recruited subjects who do not undergo surgery and/or discontinue prior to completion of the baseline visit will not be included in the targeted enrollment. The A2CPS Recruitment and Retention committee and the DIRC are continually monitoring participant enrollment and retention and data completion throughout the course of the study. The recruitment and retention committee has developed materials to facilitate recruitment including brochures, letters, physician letters, and physician videos describing the study. They have also implemented strategies to facilitate study completion and data completeness: training of research assistants in reviewing all assessments for completeness, developing rapport, expressing appreciation, and routine communication to address any challenges and obtain participant feedback, automated follow-up email reminders, assistance with transportation to data collection sites, and relationship building with physicians. The DIRC will continually monitor data completeness across measures at all sites and will notify research assistants at clinical sites of missing data. Weekly reports of missing data will be aggregated from each site and concerning patterns in missing data will trigger follow-up with the CCC training team for the research staff.

**Table 1:** A2CPS eligibility criteria.

	**Inclusion criteria**	**Exclusion criteria**
A2CPS Common Criteria	1. Provision of signed and dated informed consent form 2. Stated willingness to comply with all study procedures and availability for the duration of the study 3. Age 18–85 years	1. Patients unable to provide informed consent or unable to read/speak English or Spanish 2. Patients with known contra-indications to magnetic resonance imaging (MRI)
Knee Arthroplasty Cohort	1. Individuals diagnosed with knee osteoarthritis scheduled to undergo a single primary partial or total knee replacement; conversion of a partial to total knee replacement for mechanical failure (aseptic loosening, implant fracture, instability), wear-related complications (osteolysis, synovitis) or component malposition; or a revision of a knee replacement for mechanical failure (aseptic loosening, implant fracture, instability), wear-related complications (osteolysis, synovitis) or component malposition. All surgical approaches including robotic-controlled and muscle-sparing techniques will be included for the study.	1. Patients undergoing unilateral primary partial or total knee replacement for an inflammatory arthritic condition such as rheumatoid arthritis or osteonecrosis 2. Patients undergoing revision surgery with an infectious diagnosis involving the joint to be replaced (as this will be a 2-stage procedure) 3. Patients undergoing bilateral knee replacements, planned staged bilateral knee replacements within 3 months of each other, or are within 3 months of a prior contralateral knee replacement
Thoracic Surgery Cohort	1. Individuals who are scheduled for surgery using a thoracic approach (including thoracotomy, VATS, and robotic surgery) at any of the participating hospitals	1. Patients who have undergone prior thoracic surgery within 3 months 2. Patients undergoing a bilateral thoracic procedure 3. Patients undergoing another planned major surgery in the 6-month follow-up period

### Data Collection Procedures

#### Protocol Overview

Longitudinal assessment of the broad range of identified biomarkers (Table 2; Supplementary Table S2) will occur through a combination of in-person visits and remote assessments in all study participants (Figure 1). The full Schedule of Activities is also provided in Supplementary Table S3. Self-reported assessments are collected electronically through a variety of surveys using Research Electronic Data Capture (REDCap™) (84, 85) and/or MyDataHelps™ (RKStudio™, CareEvolution, LLC, Ann Arbor, MI) (see below for more details) (86). A2CPS includes the core psychosocial assessments and associated Helping End Addiction Long-term (HEAL) Common Data Elements (CDE) for harmonization (87). In-person visits incorporate assessment of function, pain sensitivity testing (QST), brain magnetic resonance imaging (MRI), and biospecimen collection of blood samples (omics). Additional medical data are extracted from the electronic medical record. In-person and remote assessments have been implemented for English-speaking participants, and implementation of Spanish translation and interpreter services is currently in progress.

**Table 2:** Primary biomarkers and assessments.

**Primary biomarkers**	**Assessment**
**Patient reported outcomes and behavior**	
1. General pain intensity	Brief pain intensity -whole body pain
2. Local pain intensity	Modified BPI – surgical site pain
3. Widespread body pain	Michigan Body Map
4. Acute pain trajectory following surgery	Single item assessments of daily pain and/or pain interference
5. Disability	Knee Injury and Osteoarthritis Outcome Score – 12 (Knee arthroplasty only), Danish Thoracic Surgery Questionnaire (Thoracic surgery only)
6. Perceived physical function	PROMIS Short Form v2.0 Physical Function 8b
7. Performance physical function	Five Times Sit-to-Stand, 10 Meter Walk Test (Knee arthroplasty only)
8. Movement-evoked pain	Five Times Sit-to-Stand, 10 Meter Walk Test (Knee arthroplasty only), Coughing and deep breathing (Thoracic surgery only)
9. Anxiety	General Anxiety Disorder – 7
10. Depressive symptoms	Patient Health Questionnaire – 8
11. Pain catastrophizing	Pain Catastrophizing Scale – 6
12. Fear of movement	Fear Avoidance Beliefs Questionnaire – Physical Activity
13. Sleep	PROMIS Short Form v1.0 Sleep Disturbance 6a, Sleep duration = time of sleep obtained over the past month
14. Trauma history	Adverse Childhood Experience questionnaire
15. Resilience	Pain Resilience Scale
16. Social support	PROMIS SFv2.0 Instrumental Support 6a PROMIS SFv2.0 Emotional Support 6a
17. Cognitive dysfunction	Multidimensional Inventory of Subjective Cognitive Impairment
**Omics**	
18. C-reactive protein	Proteomics/Luminex
19. Inflammatory markers – Tumor necrosis factor-alpha	Proteomics/Luminex
20. Inflammatory markers – Interleukin-6	Proteomics/Luminex
21. Inflammatory markers – Interleukin-12	Proteomics/Luminex
22. Soluble glycoprotein 130	Proteomics/Luminex
23. Catechol-O-methyltransferase haplotype (rs4680)	Genotyping Array
24. Mu-opioid receptor (rs1799971)	Genotyping Array
25. ATP binding cassette subfamily B1 (rs1045642)	Genotyping Array
26. Brain derived neurotrophic factor (rs6265)	Genotyping Array
27. Brain derived neurotrophic factor (rs1491850)	Genotyping Array
**Quantitative sensory testing (QST)**	
28. Pressure pain threshold (PPT)	Pressure algometer at surgical site
29. Temporal summation (TS)	Punctate stimulus (Neuropen) at surgical site
30. Conditioned pain modulation (CPM)	Change in shoulder PPT following noxious cold water submersion
31. Dynamic mechanical allodynia	Standardized brush (Thoracic surgery only)
**Brain imaging**	
32. Gray matter volume of medial prefrontal cortex	T1
33. Structural integrity in Am-NAc-mPFC network	Diffusion weighted imaging, white matter tractography
34. Core DMN vmPFC/CCC–NAc/ventral striatum	rsfMRI
35. Core DMN: vmPFC/CCC–somatosensory (dplNS/S1)	rsfMRI
36. Core DMN: vmPFC/CCC-anterior/middle insula	rsfMRI
37. Hub disruption	rsfMRI
38. Evoked response in neurologic pain signature	Task fMRI (pressure cuff)
39. Evoked response in fronto-striatal systems related to descending / central pain modulation and self-regulation (vmPFC, NAc)	Optimized markers (SIIPS)

*List of identified primary biomarkers and designated assessments*.

*Am, amygdala; NAc, nucleus accumbens; mPFC, medial prefrontal cortex; vmPFC, ventromedial prefrontal cortex; dlPFC, dorsolateral prefrontal cortex; CC, cingulate cortex; dpINS, dorsoposterior insula; S1 somatosensory cortex 1*.

#### Patient-Reported Outcomes

A comprehensive list of questionnaires (Table 2; Supplementary Table S2) are used to remotely collect a range of self-reported outcomes at various time-points across the study (Supplementary Table S3).

##### Pain and Fatigue

Pain is assessed using the Brief Pain Inventory (BPI) (88) and the Michigan Body Map (89). In particular, we modified the BPI to assess the worst pain intensity at the surgical site over the past 24-h with anchors of 0 (no pain) to 10 (worst pain imaginable), which was chosen as the primary study outcome. The BPI also includes the pain interference subscale (90), again specifically querying how surgical site pain has interfered with daily activity over the past 24 h with item responses ranging from 0 (does not interfere) to 10 (completely interferes). The BPI body map was replaced with the Michigan Body Map to record the number of painful body regions with persistent pain over the past 3 months (at baseline) and current pain experienced at each in-person visit. Self-reported characteristics of neuropathic pain at the surgical site is assessed with the PainDETECT Questionnaire (PD-Q) (91). The Symptom Severity Index (SSI) from the 2016 Fibromyalgia Diagnostic Criteria survey (92) is used to assess common symptoms related to chronic pain including severity of fatigue, cognitive dysfunction, and unrefreshed sleep over the past week from 0 (no problem) to 3 (severe) and incidence of lower abdomen pain, depression, and headache for at least 3 months (yes/no). In addition, fatigue and fatigue interference is assessed using the Patient Reported Outcomes Measurement Information System (PROMIS) Short Form v1.0 – Fatigue 7a (93). This seven-item measure has Likert-scale responses ranging from 1 (never) to 5 (always) with higher scores indicating greater fatigue.

##### Other Treatments and Substance Use

To screen for use of tobacco, alcohol, or illicit substances, and prescription medication misuse the Tobacco, Alcohol, Prescription medication, and other Substance use (TAPS) Part 1 and Part 2 tool (94) is used. TAPS Part 1 (4 items) screens for use over the past 12 months with item responses ranging from “never” to “daily or almost daily” while TAPS Part 2 consists of 9 additional items inquiring about use over the past 3 months with yes/no item responses. Additional items regarding smoking duration and packs per day were added.

Use of other treatments, including opioids, are assessed using items following the TAPS 1 format asking about use of common pharmacologic, rehabilitation, and integrative interventions for their pain (“never” to “daily or almost daily”). Amount of opioid use specifically is monitored at multiple time points (Supplementary Table S2) assessing specific opioids used and dosages. In addition, the Current Opioid Misuse Measure (COMM) (95, 96) is used to screen for potential risk for opioid misuse over the past month among patients reporting opioid use. The 11-item questionnaire asks patients about opioid related behaviors on a scale of 0 (never) to 5 (very often). During the immediate post-operative period (first 28 days) weekly assessments of opioid use, likeability, and side-effects are assessed. Satisfaction with pain control is assessed 6 weeks post-operatively.

##### Disability, Function, and Activity Levels

Disability is assessed using patient-specific and general measures. For the knee arthroplasty cohort, the Knee Injury and Osteoarthritis Outcome Score - 12 (KOOS-12) is a 12-item scale that assesses knee pain (4 items, never to always and none to extreme), difficulty with functional daily activities (4 items, none to extreme), and knee-related quality of life (97). For the thoracic surgery cohort, the modified Danish Thoracic Surgery Questionnaire is a 17-item scale which assesses functional impairment following thoracic surgery with item responses of 0 (“pain impairs me not at all”) to 4 (“I never do this activity due to pain”) (98, 99). General assessment of physical function for both cohorts is obtained using the PROMIS Short Form v2.0 Physical Function 8b, which contains eight questions assessing limitations in daily physical activities (response ranges: “without any difficulty” to “unable to do” and “not at all” to “cannot do”) (100, 101). Self-reported physical activity is assessed using the Rapid Assessment of Physical Activity (RAPA) consisting of 9 (yes/no) items that categorizes individuals into five physical activity levels ranging from sedentary to active (102).

##### Pain-Related Psychosocial Constructs

Several measures of pain-relevant psychosocial constructs are assessed including pain resilience, catastrophizing, kinesiophobia, and multisensory sensitivity. Pain-specific measure of resilience is assessed using the Pain Resilience Scale (PRS) (103). The PRS consists of 14 cognitive, affective and behavioral items with 0 (“not at all”) to 4 (“all the time”) responses for each question (104) where higher scores represent more resilience. Pain-related catastrophizing (highly negative appraisal of pain) is assessed using the Pain Catastrophizing Scale-6 (PCS-6) (105, 106). The PCS-6 includes 6 questions where each item is scored using 0 (“not at all”) to 4 (“all the time”) scale. In addition to the total score, three sub-scales assessing rumination, magnification and helplessness can be calculated (105, 106), where lower scores reflect less catastrophizing. Fear of movement is measured using the Fear Avoidance Beliefs Questionnaire—Physical Activity subscale (FABQ-PA) (107). The FABQ-PA subscale consists of 4-items with responses ranging from 0 (completely disagree) to 6 (completely agree) with higher scores indicating greater fear avoidance behavior. The FABQ-PA was modified replacing the term “back” pain to “knee” or “chest” pain for each respective cohort, consistent with prior studies (108–110). An 8-item General Sensory Sensitivity (GSS) scale is used to assess multisensory sensitivity to varied sensory stimuli including five external sensory stimuli (light, sound, odor, flavor, touch) and interoception (balance, nausea, heart rate) using dichotomous (yes or no) responses (111, 112) with higher scores indicating greater sensory sensitivity.

##### Depression and Anxiety

Depression and anxiety are assessed using the 8-item Patient Health Questionnaire-8 (PHQ-8) (113) and the 7-item General Anxiety Disorder (GAD-7) (114) instruments. Both the PHQ-8 and GAD-7 include questions about how often the patient experienced each item during the past 2 weeks, scored from 0 (“not at all”) to 3 (“nearly every day”). A summed score of ≥10 on PHQ-8 is considered a major depressive episode (113). A higher total score on GAD-7 represents elevated levels of anxiety (114).

##### Social Support

Two instruments are used to assess social support constructs. The quality of the supportive relationships received by the patient are assessed with the PROMIS SFv2.0 Instrumental Support 6a questionnaire (115). The patient's perceived feeling of being cared for and valued as a person is assessed using the PROMIS SFv2.0 Emotional Support 6a questionnaire (116). As in the other PROMIS questionnaires, a 5-point Likert scale ranges from “never” to “always”, and the raw scores for each patient for these two instruments will be standardized to T-scores with mean of 50 and standard deviation of 10.

##### Cognitive Function and Sleep

Cognitive function is assessed using the Multidimensional Inventory of Subjective Cognitive Impairment (MISCI), a 10-item scale that assesses varying domains of cognitive function including mental clarity, memory, attention/concentration, executive functioning, and language (117). Item responses vary from “not at all” to “very much” and “never” to “very often” with higher scores indicate a higher level of cognitive functioning. Sleep disturbance is assessed using the PROMIS Short Form v1.0 Sleep Disturbance 6a measure (118). Subjects rate their sleep quality and difficulty falling asleep over the past 14 days on a scale of 1 (not at all) to 5 (very much) with higher scores indicating greater sleep disturbance. In addition, sleep duration is assessed as the number of hours and minutes of actual sleep obtained per night during the past month (119).

##### Expectations and Perceived Change

Expectations of outcomes related to functional recovery, pain relief, and pain or complications following surgery are queried pre-operatively with 3 questions using a 0 to 10 scale. Patient's perception of pain relief following surgical intervention is assessed with the 7-item Patient's Global Impression of Change (PGIC) scale (88, 120, 121) ranging from 0 (very much improved) to 6 (very much worse) beginning 6 weeks after surgery.

##### Trajectory Items

In the acute post-surgical period from days 3 through 28, single item assessments of pain, pain interference, sleep, physical activity, medication use, and feelings of sadness, anger, and nervousness over the past 24 h are rated by participants using a 0-10 numerical rating scale. Similarly, at the 6-month follow up assessment participants complete these same items, referencing the past 7 days rather than past 24 h.

##### Other Assessments Performed at Baseline

Demographic information captured at the baseline visit includes age, sex, gender, ethnicity, race, level of education, employment status, relationship status, household income, and disability status. A modified Self-Administered Comorbidity Questionnaire (SCQ) (122) queries patients about common health problems and whether treatment is received for the related problem. Also assessed only at the baseline visit are childhood trauma and personality. The Adverse Childhood Experience questionnaire (ACE) (123) is used to retrospectively assess childhood (age 0–18 years) abuse, neglect, and household dysfunction. It has 10 items that are grouped according to adversity type. Personality is assessed using the Big Five Inventory−2 short form (BFI-2-S), a 30-item assessment used for assessing personality domains of extraversion, agreeableness, conscientiousness, negative emotionality, and open-mindedness (124). Participants rate each statement according to level of agreement from 1 (disagree strongly) to 5 (agree strongly).

##### Primary and Secondary Endpoints

Participants are sent electronic survey links to respond to a set of 6 single-item questions for 7 separate days. Participants rate their worst pain intensity (primary outcome), average pain, pain interference, sleep quality, physical activity, and pain medication intake over the past 24 h. The 7-day average of the worst pain intensity responses at the 6-month post-operative period was selected as the primary endpoint. The A2CPS biostatistics group performed a simulation based on pain ratings from other datasets to determine how to best use pain ratings from 7 sequential days. The mean daily response was chosen as the primary endpoint. Use of a continuous scale will allow for the identification of varying magnitude/severity of chronic pain which may exist post-surgically, while still allowing for secondary analyses using a dichotomous cut-point in the future. Further detail of the A2CPS statistical modeling and sample size calculations detailing the simulation study is beyond the scope of the current paper and will described elsewhere.

#### Biological Sample Collection and Processing (Omics)

Blood samples are collected from all study participants prior to surgery (baseline visit, range: 1 day−6 weeks), at the early post-operative visit (range: 3–7 weeks after surgery), and at the 3-month post-operative visit (range: ±2 weeks). Medication intake within the 24 h prior to the visit, and time of last food and stimulant intake are recorded prior to each blood draw. Two tubes, a 10 mL BD K2EDTA vacutainer (Becton-Dickinson) and a 2.5 mL BD PAXgene^®^ Blood DNA Tube, are drawn at each visit. The samples in K2EDTA tubes are centrifuged within 30 min, processed and temporarily stored at −80 degrees within 1 h locally at the clinic sites. The PAXgene samples are placed directly at−80 degrees. All samples are barcoded to link participant ID to the sample and are scanned into REDCap. Samples are shipped on dry ice from the clinical site to a central omics center (UC San Diego) for long-term storage and distribution. Sample aliquots are provided to each of the ODGC sites for genetic variant, exRNA, proteomic, lipidomic, and metabolomic analyses.

##### Genetic Variants

DNA will be extracted from 1 mL of whole blood using the QIAsymphony DSP DNA Midi Kit (Qiagen), and quantity assessed using the Qubit™ dsDNA HS Assay Kit (Thermo Fisher Scientific). Gene variants will be assessed using the Infinium Global Screening Array (GSA) BeadChip (Illumina) which combines > 650,000 multi-ethnic genome-wide content, curated clinical research variants, and quality control (QC) markers (125). In addition, the arrays will feature focused content of particular interest including ~30,000 markers associated with common psychiatric disorders (Illumina Psych Booster), ~500 additional pharmacogenomics (PGx) markers, and 5,000 custom markers selected by the A2CPS Consortium to be of particular interest in the study of chronic pain. Quality Assurance/Control will include variant call rate and sample tracking metrics included comparisons to reported sex and ancestry (126).

##### Extracellular RNAs

Extracellular RNA will be extracted from 0.5 mL of plasma using the Plasma/Serum Circulating and Exosomal RNA Purification Kit, Slurry Format (Norgen Biotek), and quality assessed using the BioAnalyzer RNA Pico Chip (Agilent). Small RNA sequencing libraries will be constructed using the NEBNext^®^ Multiplex Small RNA Library Prep Kit (NEB), pooled, size-selected using a Pippen Prep, and sequenced on an Illumina MiSeq (Nano run). The libraries will then be rebalanced and size-selected and sequenced on an Illumina NovaSeq instrument. Small RNA sequencing data preprocessing, mapping, and quality control will be performed using the ExceRpt pipeline (127). Selected miRNAs will be quantified by RT-qPCR using TaqMan Advanced miRNA Assays (Thermo Fisher Scientific).

##### Metabolomics

Metabolomics assays will be performed by teams at Wake Forest University School of Medicine and the West Coast Metabolomics Center at the University of California, Davis. Briefly, primary metabolism intermediates, particularly volatiles, non-polar and (derivatized) polar metabolites, will be targeted by the Wake Forest team using gas chromatography with high-resolution electron ionization mass spectrometry (128, 129), including targets that have been previously associated with pain phenotypes. Metabolites will be sequentially extracted and derivatized using trimethylsilylation prior to gas chromatography mass spectrometry (GCMS) analysis. Untargeted metabolomics data will be generated by high-resolution GCMS using electron impact ionization, and isotope-labeled added metabolites will be used for high confidence quantification of targets potentially related to chronic pain. Data will be processed using MS-DIAL (130) and metabolites will be identified using both in-house and public spectral libraries. Loess normalization using at least 3 external plasma QCs samples per batch will be used to normalize data to correct for batch effects. For quality assurance and system performance check, common metabolites in a quality control standard mix will be monitored to ensure confident data generation.

For biogenic metabolites such as acylcarnitines, one-carbon donor compounds, nucleotides and nucleosides, methylated and acetylated amines, di- and oligopeptides, UC Davis will use hydrophilic interaction chromatography with high-resolution tandem mass spectrometry (131). Data will be processed by MS-DIAL software (130), and compounds will be identified by MassBank.us (132) and constrained by Retip retention time software (133). Isotope labeled internal standards will ensure high confidence compound quantifications for specific targets involved in pain modulation.

##### Lipidomics

Lipidomic analyses will be completed at the West Coast Metabolomics Center at UC Davis (134) using both untargeted and targeted assays. After biphasic extraction and fractionation of polar and lipophilic compounds (135), complex lipids such as ceramides, sphingomyelins, cholesteryl esters, lyso- and diacylphospholipids, free fatty acids, and mono-, di- and triacylglycerols are separated and quantified by liquid chromatography and high-resolution mass spectrometer. Lipids are identified by our mass spectral library of 690,000 MS/MS spectra (136), in addition to accurate masses and retention time matching. Seventy-six stable isotope labeled internal standards are used for quantification, and Systematic Error Removal by Random Forest (SERRF) software is used for drift- and batch corrections from quality control pool samples (137). Specific low abundant target lipids are quantified in high-throughput LC-QTRAP 6500+ mass spectrometry. Evaluation of combined metabolomic and lipidomic profiles will be performed by chemical set enrichment statistics (138) to complement classic univariate statistics performed by the A2CPS Consortium.

##### Proteomics

Proteomic analyses will be performed in 2 phases as previously described by the team at PNNL (139–142). A discovery phase using comprehensive coverage (global) proteomics will be performed to validate previously annotated biomarker targets and to identify novel biomarker proteins and cytokines of interest. A second targeted phase will accurately quantify previously identified and novel biomarker targets using selected/multiple reaction monitoring (LC-SRM/MRM). Total protein from plasma will be isolated, enzymatically digested into peptides, and then analyzed by liquid chromatography with tandem mass-spectrometry (LC-MS/MS). For global proteomics, digested samples will be multiplexed using an isobaric labeling strategy (e.g., TMT) and analyzed in a data-dependent acquisition (DDA) mode. Targeted proteomics will be performed by optimized LC-SRM/MRM. Peptides uniquely belonging to protein biomarker targets, known as surrogate peptides, will be carefully selected to meet proteotypic and quantotypic criteria (143). Heavy isotope-labeled version of the surrogate peptides, used as standards with known concentrations, will be spiked into the digested samples and analyzed using a triple quadrupole mass spectrometer. Quantification will be based on the relative spectral intensities of endogenous peptides to heavy isotope-labeled internal standards using Skyline software (144). Quality assurance/control will include performance metrics for LC-MS/MS systems and external pooled reference plasma controls.

#### Performance-Based Function and Movement-Evoked Pain

In the knee arthroplasty cohort, physical function and movement-evoked pain (MEP) are assessed with the Five-Times Sit-to-Stand (5TSTS) Test (145, 146) and the 10-meter Walk Test (10MWT) (147, 148) following standard protocols. Time to completion is recorded for both tasks as the primary measure of function. Pain is assessed prior to and immediately following each test to assess MEP using an 11-point numerical pain rating scale (NPRS) with anchors of 0 (no pain) to 10 (worst pain imaginable). In the thoracic surgery cohort, participants are asked to take 3 deep breaths and perform 3 forceful coughs. Pain is rated prior to and immediately following each task. MEP is defined as the difference in pain with activity minus the initial resting pain.

#### Quantitative Sensory Testing

##### Pressure Pain Thresholds

Pressure Pain Thresholds (PPT) are assessed with a pressure algometer (Wagner Pain Test FPX25, Wagner Instruments, USA) applying a 1-cm^2^ rubber tip at a rate of 0.5 kgf/s. Three repetitions are performed at the surgical site (knee or chest) and three at a standard remote site (mid-deltoid of the shoulder contralateral to surgical site) (149). The average of the repetitions at each site are used as the PPT values, where higher PPTs indicate less pressure sensitivity.

##### Mechanical Temporal Summation

Temporal Summation (TS) is thought to reflect ascending pain facilitation (150); it is assessed using a punctate stimulus (Neuropen^®^, Owen Mumford, United Kingdom) applied to the skin. A single repetition is applied, followed by 10 repetitions at a rate of 1 per second, at the surgical site (knee or chest) and a standard remote site (mid-deltoid of the arm contralateral to surgical site). Pain ratings from the single stimuli and maximal pain ratings following the 10 stimuli are recorded. TS is indicated as an increase in pain ratings from repetitive noxious stimuli compared to the single stimulus (150–152).

##### Conditioned Pain Modulation

Conditioned Pain Modulation (CPM) is a psychophysical test paradigm which indirectly measures an individual's endogenous analgesia capacity (153–155). CPM assessment involves a test stimulus (PPT at the deltoid-3 repetitions) before and immediately after application of a noxious conditioning stimulus (hand immersion in a 10°C cold water bath) for 60 s (or to tolerance). CPM is indicated as an increase in PPT (less pain sensitivity) following the conditioning stimulus (154, 155).

##### Dynamic Mechanical Allodynia

Allodynia is assessed in the thoracic cohort only using a standardized brush (Somedic SENSELab Brush-05, Somedic, Sweden or equivalent) (152). Five, 4-cm brush strokes are applied at the surgical site (chest wall) and the contralateral chest wall with pain ratings recorded after each stimulation. The brush is applied with sufficient force to slightly bend the bristles 45 degrees (~200 ± 100 mN) to standardize the stimulus. The average pain ratings of the 5 repetitions at each site are used, where pain > 0 indicates allodynia.

#### Brain Imaging

Brain-imaging data will be collected using multiple sequences on 3 Tesla magnetic resonance imaging (MRI) scanners with a multi-channel (32- or 64-channel) brain coil, harmonized among 6 different imaging sites. A more detailed description of imaging methods will be provided elsewhere. Standard imaging screening will be performed at each site to ensure subject safety. Medication use over the prior 24-h and recent caffeine intake is assessed for potential influences on brain imaging. All enrolled participants are screened at each imaging site to ensure subject safety using guidelines established by the American College of Radiology (156).

Additional mood and pain items will be assessed prior to commencing scanning. The MRI protocol consists of structural MRI with T1-weighted contrast, resting-state functional MRI (rs-fMRI), task-fMRI using tonic cuff pain, and advanced diffusion imaging with multi-shell, multi-directional acquisitions. For the task-fMRI using tonic cuff pain, continuous cuff pain will be applied to the calf at (1) a subject-specific level designed to invoke a pain intensity rating of ~4/10 for 6 min and (2) at a pre-identified common pressure for all participants also for 6 min. The subject-specific cuff pressure is first identified earlier in the visit and re-checked once the participant is positioned in the scanner. The cuff is applied to the non-dominant leg for the thoracic surgery cohort and on the contralateral leg in the knee arthroplasty cohort. Two 6-min rs-fMRI runs will be performed, one before the task-fMRI runs and another immediately following them. The MRI protocol is built upon the protocol used for the Adolescent Brain Cognitive Development (ABCD) study (157, 158) but has been adapted to diverse scanner platforms across the 6 imaging sites. Before, during, and following the functional MRI runs the patient will be asked to rate any pain during the scanning procedure within their whole body, at their surgical site (knee or chest), and associated with the cuff pressure.

In addition to the above sequences, several calibration scans are performed to enable simultaneous multi-slice (SMS) imaging for faster acquisition and to reduce image distortion for better image quality. This robust MRI acquisition protocol has been harmonized on multiple scanner platforms and validated on healthy human volunteers across multiple imaging sites. To ensure the quality of the imaging data, the MRI physicists involved in the study have developed a time-efficient (~8 min) quality assurance protocol on a standard phantom. This protocol will be executed weekly throughout the study to assess both intra- and inter-site consistency in structural, diffusion-weighted, and functional imaging datasets.

#### Data Extraction From Electronic Health Record

The A2CPS Consortium will use EHR to obtain clinical and case characteristics (e.g., age, sex, height, weight, BMI, diagnosis, and surgical data). Information about the surgical procedure (e.g., procedure name, Current Procedural Terminology code, surgeon, laterality, duration, and anesthesia) will be extracted. Procedural anesthesia information will include American Society of Anesthesiology physical status, the primary anesthesia type, duration of anesthesia and medications administered during anesthesia care. Post-procedural information will be extracted including pain assessments, medications, and length of stay. Opioid medications will be converted to milligram morphine equivalents. For the knee arthroplasty cohort, the implant name, manufacturer, and number will be obtained. In the thoracic surgery cohort, data will be obtained from the Multicenter Perioperative Outcomes Group (MPOG) (159) and Michigan Society of Thoracic and Cardiovascular Surgeons (MSTCVS) databases; these sources allow additional structured information on medical history and 30-day postoperative outcomes to be included. Use of EHR derived data is included in study participant informed consent. Health Insurance Portability and Accountability Act (HIPAA) rules, other relevant federal or state laws, and local institutional requirements will be followed, as applicable.

### Study Endpoints

Our primary endpoint, chronic pain at 6 months following surgery, will be operationally defined as worst pain in the last 24 h. This will be assessed on a 0–10 numeric rating scale (NRS) for 7 days, with the average value used as the endpoint. Additional secondary endpoints include measures of dysfunction (disability) and opioid use at the 6-month follow-up. Exploratory endpoints assessed at the 6-month follow-up include: pain interference; worst pain intensity at a single time at 6 months; use and misuse of opioids; perceived global impact of change; depressive symptoms; anxiety; and sleep quality. Similar items will be assessed at 12 months through remote electronic query as an optional follow-up.

### Data Management and Analyses

#### Data Management

Data collected for this study will be analyzed and stored by the DIRC at the Texas Advanced Computing Center (TACC) based at the University of Texas. A data collection system using REDCap has been set up to collect patient-reported outcomes and researcher-driven data from the MCCs. For MCC1, patient surveys are sent out via REDCap at regularly scheduled time periods dictated by the study protocol. MCC2 has made the surveys available within the MyDataHelps app, allowing participants to complete the surveys directly using a smartphone app. The app data is automatically transferred daily into REDCap. Both MCCs use REDCap to fill out comprehensive information about the functional testing, QST, blood draws, and brain-imaging sessions. In addition, any protocol deviations are recorded in REDCap. All study participant research data is transmitted to and stored by the DIRC. This includes imaging data, electronic health records, processed omics data, and information collected in REDCap. Individual participants and their research data are identified by a unique study identification number and a universally unique identifier (UUID). To facilitate generalizability and consistency among studies, the HEAL CDE are used to code all data. Any missing data or data anomalies will be communicated to the center(s) for clarification/resolution.

#### Sample Size Determination

The appropriate sample size was determined both considering hypothesis testing and prediction as a primary analysis. For hypothesis testing, power was estimated using logistic regression models for binary biomarkers and *t*-tests for continuous biomarkers with effect sizes derived from existing literature under each of the following conditions: Type I Error (α) = 0.01, 0.05; Sample Size (*N*) = 1,000, 1,400, 1,800; and Transition Rate (to chronic pain) = 15%, 25%. For each combination of alpha, sample size, and transition rate we created a summary of which biomarkers would meet a threshold of at least 80% power. For prediction, sample size was determined based on controlling the prediction error bound at 0.025 assuming a transition rate of 25%. The initial sample size recommendation was 1,800 participants per cohort, allowing for a total number of up to 40 primary biomarkers. However, preliminary feasibility analyses after the first 100 participants have led to adjustments to our priori sample size calculations from a more conservative sample of 1,800 to 1,400, with minimal sample size estimates of 1,000. Thus, we are targeting ~1,400 participants per cohort to cover attrition and missing data.

#### Planned Statistical Analyses

##### Primary Analyses

The primary analysis involves the use of a candidate approach to examine whether the putative biomarkers across multiple domains (i.e., clinical, biospecimen, psychosocial, and brain structure/function) individually predict susceptibility or resilience to the development of chronic pain at 6 months after an acute painful event. The data will be analyzed using a predictive modeling approach with k-fold cross validation. Multiple regression will be used to evaluate how each of the pre-selected biomarkers of interest individually account for unique variance in predicting the transition from acute to chronic pain. The model will include potential confounders [e.g., differences across sites/batch effects in training sample size and head movement (for brain imaging)] and baseline characteristics (e.g., sex, age, socio-economic status, baseline pain). Significance will be assessed using the Wald test for the regression coefficient and effect size using a 0.05 significance level. All tests will be two-sided, unless a one-sided test is specifically called for by the biomarker (e.g., reduced volume of medial prefrontal cortex). Given the focus on prediction, multiplicity adjustment is not necessary in this phase. As each cohort corresponds to a different type of surgery, we will perform analyses both separately and combined.

##### Secondary Analyses

Secondary analyses involve the development of a biosignature(s) using the candidate primary biomarkers evaluated individually in the primary approach, to determine if combinations of biomarkers improve the prediction of transition from acute to chronic pain after an acute painful event. We will analyze the data using a predictive modeling approach. We will use a machine learning approach (e.g., random forest) to select the most predictive variables. Models will also investigate whether the relationships between biomarkers and our outcome is mediated by baseline characteristics. Methods such as random forest are ideal in this setting as they are particularly designed to find interactions between variables. Significance will be assessed using a Bootstrap procedure at the 0.05 significance level. Surgical cohort will be included as a variable to evaluate its interaction with all the predictors. We will assess the variability accounted for on the primary outcome by each combination of biomarkers. Within each type (e.g., genomics, brain imaging, behavioral) we can conduct a complete search and enumeration of all models. Variables can then be combined across types. We will further focus on finding combinations that require as few data collection modalities as possible.

##### Exploratory Analyses

For the exploratory analysis, we will use a discovery-validation approach to define novel putative biomarkers and biosignatures across multiple domains that predict the susceptibility or resilience to development of chronic pain at 6 months. We will use multivariate predictive modeling to identify latent variables within sets of measures with multi-objective machine learning-based optimization of predictive accuracy, specificity to pain, generalizability across cohorts, and model reproducibility. Techniques include feature selection and feature engineering combined with penalized regression/classification, ensemble classifiers, and deep learning/convolutional neural networks where applicable. Outcomes will go beyond pain ratings alone to include latent trajectories of pain intensity and interference, chronic opioid use, physical and emotional function, and other domains. We will adopt a model-building practice of comparing simpler, more interpretable models with more complex ones, adding complexity only when essential and warranted by the data, and developing models to be comprehensible and usable by a broad audience. Prospective validation datasets will be separated and sequestered in advance. Training samples will be balanced on covariates using propensity score matching, ensuring that models are not driven by confounds. A systematic evaluation of processing, scaling, algorithms, and tuning parameters will be performed within k-fold cross-validation loops, leading to rigorous comparisons across methods and options, with stochastic optimization for intractably large model sets. Validation datasets will be tested only on final, optimized models with “locked down” parameter estimates, and evaluated for performance on multiple objective functions: sensitivity, specificity, and model complexity.

##### Interim Analyses

An initial feasibility assessment is planned after approximately the first 100 enrolled participants (1) to assess for any data collection concerns (baseline thru 3-month missing data analyses) and (2) to assess preliminary results following completion of the study (6 months after surgery/acute pain assessment). This will be done to better inform the risk prediction sample size calculations. In addition, a futility analysis will be performed for primary biomarkers and the primary study outcome after 50% of participants are completed. This futility analysis will assess whether the transition rate to chronic pain is adequate for the final analysis of the data.

#### Data Sharing

At the end of the study, all study databases will be archived by the DIRC. The de-identified datasets will be transmitted to and stored at the NIH HEAL Data Ecosystem, for use by other researchers including those outside of the study. With the participant's approval and as approved by the central internal review board (cIRB), de-identified biological samples will be stored at the HEAL Biorepository [Early Phase Pain Investigation Clinical Network (EPPIC-Net) at New York University] to be used to further research the causes of chronic pain and to improve treatment. The HEAL Data and Biorepositories will also be provided with a code-link that will allow linking the biological specimens with the phenotypic data from each participant, maintaining the blinding of the identity of the participant. In addition, links between the unique study identification number and the globally unique identifier will be maintained in the form of a registry by the DIRC for an undetermined time pending long-term resources. Permission to transmit data to the data repository is included in the informed consent. This dataset will serve as a resource for the investigators to evaluate a wide range of scientific inquiries, a priority for the A2CPS program.

### Regulatory and Quality Control

#### Quality Assurance and Control

The A2CPS CCC and DIRC collaboratively provide quality oversight for all Consortium activities conducted at the MCCs and ODGCs. To ensure collection of high-quality data, we standardized data-collection procedures across sites, documented in our manual of procedures. All study personnel involved in data collection completed training and certification for relevant study procedures, including recruitment and consent, PROs, function, biospecimen collection, QST, and brain imaging. All Consortium personnel establish proficiency on standard operating procedures (SOPs), good clinical practice (GCP), the protection of human subjects, and standardized data collection and entry procedures. Adherence to established study protocols is necessary to obtain rigorous and reliable data collection longitudinally and across clinical sites.

The DIRC is responsible for developing, testing, and managing clinical data management activities, as required. All study data is collected by systems that comply with all applicable guidelines regarding patient confidentiality and data integrity (REDCap and MyDataHelps). The study data entry and study management systems used by clinical sites and by the DIRC research staff is secure and password protected.

#### Central Internal Review Board and Safety Monitoring

The A2CPS utilizes the University of Iowa as the cIRB for all study review. Documented approval from the A2CPS cIRB (IRB ID#: 201905783) and all necessary reliance agreements were obtained for all participating centers prior to commencing subject recruitment and data collection. The date of the initial cIRB approval to recruit subjects at the first site was October 5, 2020, with subsequent amendment approvals to add the additional sites. All participants are provided detailed descriptions of the study purpose, procedures and risks provided, and confirmation their decision to participate or withdrawal from the study in no way alters their medical care by clinical research staff at each site. Documentation of informed consent is completed electronically, in person or virtually prior to starting data collection. During the course of the study, an individual participant can choose to withdraw from the study at any time or withdraw consent to have biological specimens stored for future research. However, withdrawal of consent regarding biosample storage may not be possible after the study is completed and de-identified data has been transferred to the HEAL repositories.

#### Data Safety Monitoring Plan

Safety oversight for the A2CPS follows a DSMP with regular study review by external medical monitor. We assess and monitor safety in relation to participation in research study procedures. Adverse events are defined as those related to study procedures only, i.e., infection of blood draw site or claustrophobia during MRI, not new or worsened medical conditions, as this is an observational study and not a clinical trial. However, any other participant issues will be reported as unanticipated problems (UAPs). The CCC will monitor clinical sites to ensure compliance with study data collection procedures and data management requirements using both on-site and remote monitoring procedures.

## Discussion

The A2CPS is a large multisite project that involves a diverse group of scientists. Our collaborative approach included group discussions with representation from all centers and the NIH staff to make decisions by consensus. Efforts were made to balance competing concerns of obtaining the most complete participant phenotyping while minimizing participant burden. The Consortium launched in September 2019 with the CCC, DIRC, ODGCs and only one MCC. The second MCC was brought into the Consortium in August 2020, resulting in unique challenges to harmonize data-collection procedures and consider inclusion of additional biomarkers. The integration of MCCs at different timepoints ultimately delayed the start of data collection for MCC1 and placed the MCCs on different study timelines.

The A2CPS has further experienced challenges associated with COVID-19, leading to adaptations in processes required to complete study design, implement protocols, and initiate recruitment. The Consortium has adapted to having all-virtual meetings to facilitate collaboration and continued study development. Training in study procedures were transitioned from onsite training to virtual training. There were unanticipated delays in regulatory approvals due to increased staff workloads due to COVID-19 protocol changes and increased administrative burden. Additionally, elective surgeries and recruitment were paused at times due to pandemic mitigation strategies. The Consortium also experienced delays in obtaining blood sample collection materials.

Despite these challenges, preliminary checks at both MCCs show high data completion rates for surveys and assessments, with neuroimaging, omics, and psychophysical assessments satisfying established quality control metrics. Once completed, the data generated from the A2CPS program will help identify risk and resilience biomarkers and biosignatures of the transition from acute to chronic pain. These results will be available to the scientific community for further data mining, and provide the potential to inform future clinical trials, identify novel therapeutic targets, advance personalized acute pain treatment strategies, thereby transforming the management of acute pain events.

In summary, the A2CPS brings together a consortium of experts in pain science, imaging, QST, omics, and the management of large clinical studies from multiple sites across the United States. This multisite observational study provides the ability to comprehensively phenotype a large cohort of individuals across multiple biopsychosocial domains in a relatively short time. The ultimate goal is to identify and validate novel biomarker candidates that can serve as a part of a final biosignature for predicting the susceptibility or resilience to the development of chronic post-surgical pain. Investigation of previously identified and novel biomarkers in a large cohort will help identify which biomarkers provide greatest prognostic value in a larger cohort and may help direct the focus of clinically relevant biomarkers that can be implemented in daily practice. Results from this study may not be generalizable to all surgical and chronic pain cohorts as contributing biopsychosocial factors may vary by diagnosis. Additionally, recruitment of a diverse population is inherently challenging, and the sample recruited for this observational study may not be representative of individuals from all races, ethnicities, and sociodemographic backgrounds. Beyond stated Consortium aims, A2CPS will provide the broad research community with a valuable dataset to drive future hypothesis-driven research.

### Study Status and Progress

Recruitment started in 2021 and is ongoing at both MCCs according to established procedures in A2CPS Protocol #6.0 (February 14, 2022). As of January 2022, a total of 337 individuals (276 knee replacement, 61 thoracic surgery) have been enrolled in the study with 288 completing surgery (238 knee replacement, 50 thoracic surgery), 56 reaching the 6-month assessment (54 knee replacement, 2 thoracic surgery) and no participants at the 12-month assessment. A brief summary of completion rates by biomarker domains is provided in Supplementary Table 4. This study is estimated to be completed 48 months from the time of initial participant enrollment to completion of the final participant.

## Ethics Statement

This study was approved by the University of Iowa Institutional Review Board, serving as the central IRB. All patients/participants provided their written informed consent prior to study participation.

## Author Contributions

The manuscript was drafted by GB, LF-L, EB, DCl, CB, JB, RM, XZ, ML, DCo, LL, KF, KJ, MO, CL, AS, and PD, and all authors reviewed and provided edits to the manuscript. All authors contributed to the study conception and design and approved the final manuscript.

## Funding

The A2CPS Consortium was supported by the National Institutes of Health Common Fund, which was managed by the OD/Office of Strategic Coordination (OSC). Consortium components include: Clinical Coordinating Center (UO1NS077179), Data Integration and Resource Center (UO1NS077352), Omics Data Generation Centers (U54DA049116, U54DA049115, U54DA09113), and Multisite Clinical Centers: MCC 1 (UM1NS112874) and MCC 2 (UM1NS118922). Postdoctoral support for GB provided by the National Institutes of Neurological Disease and Stroke (NINDS) of the NIH under Award Number: U24NS112873-03S2. Training and research support for DCo provided by the NHLBI under Award Number: K08 HL159327.

## Author Disclaimer

The content is solely the responsibility of the authors and does not necessarily represent the official views of the National Institutes of Health.

## Conflict of Interest

The authors declare that the research was conducted in the absence of any commercial or financial relationships that could be construed as a potential conflict of interest.

## Publisher's Note

All claims expressed in this article are solely those of the authors and do not necessarily represent those of their affiliated organizations, or those of the publisher, the editors and the reviewers. Any product that may be evaluated in this article, or claim that may be made by its manufacturer, is not guaranteed or endorsed by the publisher.

## Abbreviations

5TSTS: Five-Times Sit-to-Stand
10MWT: 10-meter Walk Test
A2CPS: Acute to Chronic Pain Signatures
ABCD: Adolescent Brain Cognitive Development
ACE: Adverse Childhood Experience
BFI-2-S: Big Five Inventory-2 Short Form
BPI: Brief Pain Inventory
CCC: Clinical Coordinating Center
CDE: Common Data Elements
cIRB: Central Institutional Review Board
CPM: Conditioned pain modulation
CPSP: Chronic post-surgical pain
COMM: Current Opioid Misuse Measure
DIRC: Data Integration and Resource Center
DSMP: Data Safety Monitoring Plan
EPPIC-Net: Early Phase Pain Investigation Clinical Network
FABQ-PA: Fear Avoidance Beliefs Questionnaire &#8211
Physical Activity
fMRI: Functional magnetic resonance imaging
GAD-7: General Anxiety Disorder &#8211
7
GCMS: Gas chromatography mass spectrometry
GCP: Good clinical practice
GLP: Good laboratory practice
GMP: Good manufacturing practices
GSA: Global Screening Array
GSS: General Sensory Sensitivity
HEAL: Helping to End Addiction Long-term
KOOS-12: Knee Injury and Osteoarthritis Outcome Score - 12
LC-MS/MS: Liquid Chromatography with tandem mass spectrometry
MCC: Multisite Clinical Center
MISCI: Multidimensional Inventory of Subjective Cognitive Impairment
MRI: Magnetic resonance imaging
NCATS: National Center for Advancing Translational Sciences
NIH: National Institutes of Health
NPRS: Numerical Pain Rating Scale
NRS: Numerical Rating Scale
ODGC: Omics Data Generation Center
PCS-6: Pain Catastrophizing Scale &#8211
6
PD-Q: PainDETECT Questionnaire
PGIC: Patient's Global Impression of Change
PGx: Pharmacogenomics
PHQ-8: Patient Health Questionnaire &#8211
8
PPT: Pressure Pain Threshold
PROMIS: Patient-Reported Outcomes Measurement Information System
PRS: Pain Resilience Scale
QC: Quality control
QST: Quantitative sensory testing
RAPA: Rapid Assessment of Physical Activity
REDCap: Research Electronic Data Capture
rs-fMRI: Resting-state fMRI
SCQ: Self-Administered Comorbidity Questionnaire
SOP: Standard operating procedure
SSI: Symptom Severity Index
TACC: Texas Advanced Computing Center
TAPS, Tobacco, Alcohol, Prescription medication: and other Substance use
TS: Temporal summation
UAP: Unanticipated problem
UUID: Universally unique identifier
VATS: Video-assisted thoracic surgery.
