# Supplementary material for: Multi-Site Observational Study to Assess Biomarkers for Susceptibility or Resilience to Chronic Pain: The Acute to Chronic Pain Signatures (A2CPS) Study Protocol
Source: Front Med (Lausanne). 2022 Apr 25;9:849214. doi: 10.3389/fmed.2022.849214 (PMC9082267; doi:10.3389/fmed.2022.849214)
Supplement: Supplementary file 1 [file Data_Sheet_1.pdf]

Supplementary Material

Supplementary Table 1. A2CPS Consortium Sites & Roles. Each consortium site consists of institutions with designated roles to fulfill A2CPS objectives.

A2CPS Consortium Site	Institutions	Role
**Clinical Coordinating Center (CCC)**	• University of Iowa	Serve as the hub for MCCs. The CCC will support study design, efficiency, progress, and quality, will coordinate/monitor study implementation across the clinical sites, and will lead the consortium in developing and implementing protocols and standards
**Data Integration and Research Center (DIRC)**	• Johns Hopkins University<br>• Dartmouth University<br>• Texas Advanced Computing Center	Integrate the efforts of all funded components of the A2CPS and serve as a community-wide nexus for protocols, data, assay and data standards, and other resources generated by the A2CPS Program
**Omics Data Generation Centers (ODGCs)**	• University of California, San Diego<br>• Wake Forest University<br>• Pacific Northwest National Laboratory<br>• West Coast Metabolomics Center at University of California, Davis<br>• University of Pittsburgh	Use cutting edge technologies to perform omics analyses (e.g., metabolomic, lipidomic, proteomic, genetics, extracellular RNA) of body fluids collected by the Acute to Chronic Pain Signatures (A2CPS) Consortium
**Multisite Clinical Centers (MCCs)**	MCC1:<br>• Rush University Medical Center<br>• University of Chicago<br>• University of Illinois at Chicago<br>• NorthShore University Health System<br>MCC2:<br>• University of Michigan<br>• Wayne State University<br>• Spectrum Health*<br>• St. Joseph Mercy Health System (Ann Arbor)<br>• Henry Ford Health System (Detroit, Macomb, Jackson)	Implement the enrollment and multimodal longitudinal assessment of a large cohort of acute peri-operative pain patients (total knee arthroplasty and thoracic surgery) to identify a biosignature for resilience to and the transition from acute to chronic pain

*Not fully activated as of February 2022

Supplementary Table 2. Secondary Biomarkers and Assessments. List of identified secondary biomarkers and designated assessments.

Secondary Biomarkers	Assessment
**Subject Characteristics**	
1. Age	Demographics questionnaire
2. Sex/gender	Demographics questionnaire
3. Ethnicity/race	Demographics questionnaire
4. Education	Demographics questionnaire
5. Relationship status	Demographics questionnaire
6. Income	Demographics questionnaire
7. Disability insurance	Demographics questionnaire
8. Co-morbidities	Self-Administered Comorbidities Questionnaire
9. Opioid use	Opioid use items
10. Opioid likeability	Opioid likeability and side-effect items
11. Opioid or other substance misuse	Tobacco, Alcohol, Prescription medication, and other Substance use (TAPS)<br>Current Opioid Misuse Measure (COMM)
12. Other pharmacologic treatments	Other treatments questionnaire
13. Other non-pharmacologic treatments	Other treatments questionnaire
14. Smoking	TAPS + smoking duration and packs per day
15. Surgery and hospital stay details	Electronic health record data
**Patient Reported Outcomes & Behavior**	
16. Pain duration	Demographics questionnaire
17. Pain impact/quality of life (surgery specific)	Knee Injury and Osteoarthritis Outcome Score – 12<br>Danish Thoracic Surgery Questionnaire
18. Trajectory assessments	Single item daily assessments of sleep, physical activity, medication, sadness, anger, and nervousness during post-surgical period
19. Pain interference	Brief Pain Inventory – Interference scale<br>Acute trajectory following surgery
20. Physical activity level	Rapid Assessment of Physical Activity
21. Personality – neuroticism	Big Five Inventory–2 short form
22. Personality – agreeableness	Big Five Inventory–2 short form
23. Personality – conscientiousness	Big Five Inventory–2 short form
24. Personality – openness	Big Five Inventory–2 short form
25. Personality – extraversion	Big Five Inventory–2 short form
26. Multisensory sensitivity	General Sensory Sensitivity – 8
27. Expectation	Single item questions, 0-10 NRS
28. Global impression of change	Patient’s Global Impression of Change (PGIC)
29. Fatigue	PROMIS Short Form v1.0 – Fatigue 7a
30. Neuropathic pain symptoms	PainDETECT
31. Acute pain	EHR data, acute Michigan Body Map
**Omics**	
32. Interferon-gamma	Proteomics/Luminex
33. Interleukin-17	Proteomics/Luminex
34. Interleukin-1beta	Proteomics/Luminex
35. Heparin-binding epidermal growth factor	Proteomics/Luminex
36. Monocyte chemoattractant protein-1 (MCP-1/CCL2)	Proteomics/Luminex

37. Cyclooxygenase-2	Proteomics/Luminex
38. Interleukin-4	Proteomics/Luminex
39. Interleukin-5	Proteomics/Luminex
40. Interleukin-10	Proteomics/Luminex
41. Interleukin-13	Proteomics/Luminex
42. Brain derived neurotrophic factor	Proteomics/Luminex
43. Nerve growth factor	Proteomics/Luminex
44. Leptin	Proteomics/Luminex
45. Adiponectin	Proteomics/Luminex
46. miR-223-3p	exRNA
47. Interleukin-6 (rs2069845)	SNP Array
48. Interleukin-13 (rs1295686)	SNP Array
49. Tumor necrosis factor-alpha (rs1800610)	SNP Array
50. Serotonergic signaling pathway (rs9316233, rs4776783, rs12439516, rs2276008, rs6928, rs3813928)	SNP Array
51. T-cell receptor pathway (rs10500205, rs216535, rs306083, rs3797739, rs2070995, rs3756612, rs815815, rs790250)	SNP Array
52. Voltage-gated K channel subunit KV9.1 (rs734784)	SNP Array
53. Nuclear receptor subfamily-3 group-C member 1 (rs2963155)	SNP Array
54. GTP cyclohydrolase 1 (rs998259)	SNP Array
55. Palmitoylethanolamide (NAE 16:0)	Lipidomics
56. 2-Arachidonoylglycerol	Lipidomics
57. Sphingomyelin (d18:1/16:0)	Lipidomics
58. Ceramide (d18:1/16:0)	Lipidomics
59. Phosphoinositol (18:0/20:4)	Lipidomics
60. Threonic acid	Metabolomics
61. Nonanoic acid	Metabolomics
62. Hypoxanthine	Metabolomics
63. Inosine	Metabolomics
64. Kynurenic acid	Metabolomics
**Quantitative Sensory Testing**	
65. Pressure pain threshold	Pressure algometer at shoulder
66. Temporal summation	Punctate stimulus Neuropen at shoulder
67. Cuff pain sensitivity	Pressure cuff and pain rating
**Brain Imaging**	
68. Gray matter density	T1
69. Gray matter morphometry	T1 gray matter
70. Gray matter volume of hippocampus	T1
71. Gray matter volume of amygdala	T1
72. Node degree Hipp, R dlPFC	rsfMRI
73. Lateral thalamus - PAG connectivity	rsfMRI
74. FA in superior longitudinal fasciculus (fronto-parietal), internal and external capsule	Diffusion weighted imaging, white matter tractography
75. Local pattern responses in nociceptive regions	Task fMRI (pressure cuff)
76. Context-related local pattern responses	Task fMRI (pressure cuff)
77. Response to pain offset in reward systems	Task fMRI (pressure cuff)

frontiers

Supplementary Material

Supplementary Table 3. Schedule of Activities. Detailed list and schedule of assessments over the course of the study.

Procedures		Consent	Baseline surveys**	Baseline Clinic Visit	3d pre-op**	Acute phase**	6-wks post-op ** surveys	6-wks post-op clinic visit	3-mo post-op ** surveys	3-mo post-op clinic visit	6-mo post-op ** surveys	12-mo post-op ** surveys
**Informed Consent**		X										
**Demographics**			X									
**Co-morbidities Questionnaire**			X									
**Substance use: TAPS-1 & 2**			X								X	
**Opioid Use:**	**Other Treatments**		X				X		X		X	
	**Opioid Use**		X			q7d	X		X		X	
	**COMM**		X			d28			X		X	
	**Likeability & Side Effects**					d7,28						
	**Pain control satisfaction**						X					
**Perceived Change:**	**PGIC**						X		X		X	
**Pain:**	**Pain & Interference (BPI)**		X				X		X		X	X
	**MI Body Map (Chronic)**		X				X		X		X	X
	**MI Body Map (Acute)**			X						X		
	**Symptom Severity Index**		X				X		X		X	
	**PainDETECT**		X				X		X		X	
**Function – Diagnosis Specific:**	**(KOOS)**		X#						X#		X#	
	**(Mod Danish Thoracic Survey)**		X+						X+		X+	
	**General Physical Function (PROMIS PF8)**		X						X		X	
	**Physical Activity (RAPA)**		X						X		X	
**Sleep:**	**PROMIS Sleep**		X				X		X		X	
**Pain-related:**	**Resilience (PRS)**		X									
	**Catastrophizing (PCS6)**		X				X		X			
	**Kinesiophobia (FABQ-PA)**		X				X		X			
	**Multisensory (GSS8)**		X								X	

Procedures		Consent	Baseline surveys**	Baseline Clinic Visit	3d pre-op**	Acute phase**	6-wks post-op ** surveys	6-wks post-op clinic visit	3-mo post-op ** surveys	3-mo post-op clinic visit	6-mo post-op ** surveys	12-mo post-op ** surveys
**Other:**	**Depression (PHQ-8)**		X				X		X		X	
	**Anxiety (GAD-7)**		X				X		X		X	
	**Social & Instrumental Support-(PROMIS)**		X				X		X			
	**Expectation-3**				X							
	**Trauma Hx (ACE)**		X									
	**Personality (BF-S-2)**		X									
	**Cognitive Dysfunction (MISCI)**		X				X		X		X	
	**Fatigue (PROMIS)**		X				X		X		X	
**Trajectory survey items**						X d3-25					X x7d	X 1d
**"Omics":**	**Blood draw**			X				X		X		
	**Meal, caffeine, meds survey**			X				X		X		
**Function Tests:**	**5TSTS, 10m walk**			X#						X#		
	**Coughing/deep breathing**			X+		X+ d7,14, 21,28	X+		X+	X+	X+	
**QST:**	**PPTs**			X						X		
	**TS**			X						X		
	**CPM**			X						X		
	**Allodynia**			X+						X+		
**MRI**	**survey: pain, mood**			X						X		
	**T1, DWI**			X						X		
	**fMRI: Rest /cuff x2 /rest**			X						X		
	**Visit satisfaction assessment**			X								

Acute phase = days 3 to 28 post-op, **= remote assessment, # = knee arthroplasty cohort only, + = thoracic surgery cohort, q = every, d = days

Supplementary Material

Supplementary Table 4. Data Completion Rates by Biomarker Domain. Completion rates represented by number completed to number expected as of January 2022.

Biomarker	Knee Arthroplasty			Thoracic Surgery		
	Baseline	3 Months	6 Months	Baseline	3 Months	6 Months
Surveys	233 / 237	142 / 154	61 / 61	50 / 50	22 / 22	2 / 2
Functional Assessments	222 / 235	95 / 148	N/A	50 / 50	18 / 21	N/A
Quantitative Sensory Testing	224 / 235	94 / 148	N/A	49 / 50	18 / 21	N/A
Imaging	180 / 235	71 / 90	N/A	40 / 50	11 / 21	N/A
Biospecimen Sample	220 / 235	91 / 148	N/A	48 / 50	18 / 21	N/A
